# Atacicept in relapsed/refractory multiple myeloma or active Waldenström's macroglobulinemia: a phase I study

**DOI:** 10.1038/sj.bjc.6605241

**Published:** 2009-09-29

**Authors:** J-F Rossi, J Moreaux, D Hose, G Requirand, M Rose, V Rouillé, I Nestorov, G Mordenti, H Goldschmidt, A Ythier, B Klein

**Affiliations:** 1 CIC-Biothérapie BT 509, CHU Montpellier, Montpellier, France; 2 INSERM, U847, Montpellier, France; 3 Medizinische Klinik und Poliklinik V, Universitätsklinikum Heidelberg, INF410, Germany; 4 Université Montpellier1, France; 5 Department of Haematology and Clinical Oncology, CHU Montpellier, France; 6 Clinical Development, ZymoGenetics Inc., Seattle, WA, USA; 7 Clinical Development, Merck Serono S.A. (an affiliate of Merck KGaA, Darmstadt, Germany), Geneva, Switzerland

**Keywords:** atacicept, multiple myeloma, Waldenström's macroglobulinemia, BLyS, APRIL

## Abstract

**Background:**

Advanced multiple myeloma (MM) and Waldenström's macroglobulinemia (WM) are incurable B-cell malignancies. This is the first full clinical report of atacicept, a fusion protein that binds to and neutralises the B-cell survival factors, B-lymphocyte stimulator (BLyS) and A proliferation-inducing ligand (APRIL), in MM and WM.

**Methods:**

In this open-label phase-I study, 16 patients with advanced disease (12 MM, 4 WM) received one cycle of five once-weekly subcutaneous injections of atacicept (2, 4, 7 or 10 mg kg^−1^). Patients with stable disease after cycle 1 entered an extension study (either two additional cycles (2, 4 and 7 mg kg^−1^ cohorts) or 15 consecutive weekly injections of atacicept 10 mg kg^−1^).

**Results:**

Atacicept was well tolerated, systemically and locally; the maximum tolerated dose was not identified. Of 11 patients with MM who completed initial treatment, five patients were progression-free after cycle 1 and four patients were progression-free after extended therapy. Of four patients with WM, three patients were progression-free after cycle 1. Consistent with atacicept's mechanism of action, polyclonal immunoglobulin isotypes and total B cells were reduced. Bone-marrow density, myeloma cell numbers and plasma concentrations of soluble CD138 also decreased.

**Conclusion:**

Atacicept is well tolerated in patients with MM and WM, and shows clinical and biological activity consistent with its mechanism of action.

Multiple myeloma (MM) is the second most prevalent blood cancer, accounting for 10% of haematological malignancies. The median age at diagnosis is 68 years and the incidence increases with advancing age (Ferlay *et al*, 2004). Among patients treated with conventional chemotherapy, the median survival is about 42 months (Bladé *et al*, 1994). In patients younger than 65 years, high-dose chemotherapy supported by autologous stem cell transplantation increases the median survival to 60 months (Harousseau and Moreau, 2007). Despite recent innovations in approaches to therapy, options remain limited for the majority of patients.

Waldenström's macroglobulinemia (WM) is a B-cell malignancy, which results from a clonal proliferation of lymphocytes and plasma cells that produce monoclonal immunoglobulin (Ig) M, and our understanding of this clinico-pathological entity has improved in recent years (Vitolo *et al*, 2008; Dimopoulos *et al*, 2009). The median age at presentation is 63 years (Dimopoulos *et al*, 2000). The Fourth International Workshop on WM reviewed treatment guidelines for patients with WM who require systemic therapy; current primary treatments include alkylating agents, nucleoside analogues and immunotherapies, as single agents and in combination regimens (Dimopoulos *et al*, 2009). The need for and development of new therapies for patients with WM was further discussed at the recent Fifth International Workshop on WM (Treon *et al*, 2009).

B-lymphocyte stimulator (BLyS, CD257, also known as B-cell activating factor (BAFF)) (Schneider *et al*, 1999) and A proliferation-inducing ligand (APRIL, CD256) stimulate the growth of primary myeloma cells (Moreaux *et al*, 2004; Novak *et al*, 2004). BLyS and APRIL share two receptors, trans-membrane activator and CAML interactor (TACI, CD267) (Von Bulow and Bram, 1997) and B-cell maturation antigen (BCMA, CD269) (Gras *et al*, 1995); BLyS also binds to a third receptor, BAFF-R (also known as BR3, CD268) (Thompson *et al*, 2001). Most myeloma cell lines and primary myeloma cells express one or more of these receptors for BLyS (Novak *et al*, 2004; Moreaux *et al*, 2007, 2009) and also aberrantly express BLyS and APRIL mRNA (Moreaux *et al*, 2004). BLyS and APRIL are also produced in large amounts in the tumour bone marrow environment, primarily by myeloid cells, monocytes and osteoclasts (Moreaux *et al*, 2005; Abe *et al*, 2006; Tai *et al*, 2006; Yaccoby *et al*, 2008). Exogenous BLyS and APRIL act as potent survival factors for primary myeloma cells cultured within the bone marrow microenvironment (Moreaux *et al*, 2004, 2009). Similar patterns of BLyS expression and tumourigenic activity have been observed in patients with WM (Elsawa *et al*, 2006).

Atacicept is a fusion protein composed of the human IgG Fc portion and the extracellular, ligand-binding portion of the TACI receptor, which neutralises both BLyS and APRIL (Gross *et al*, 2000, 2001). The addition of atacicept to primary myeloma cells abrogates proliferative effects and induces apoptosis (Moreaux *et al*, 2004; Abe *et al*, 2006).

The purpose of this investigation was to assess the overall safety and tolerability of atacicept in patients with refractory or relapsed MM or progressive WM, and to evaluate the overall clinical-benefit rate, pharmacokinetics, pharmacodynamics and immmunogenicity of atacicept in this population.

## Materials and methods

### Study design and interventions

We report the results of two studies conducted in one centre in France. The protocols were developed in collaboration with, and approved by, an independent ethics committee, and the studies were carried out in accordance with the Declaration of Helsinki, the standards of the International Conference on Harmonization, Guideline for Good Clinical Practice and local regulations. Patients gave written informed consent for participation in each study. This study was initiated in November 2004 and at that time was not registered in a clinical trial registry.

Adults (⩾18 years of age) with confirmed relapsed and/or refractory MM or progressive WM were recruited into an exploratory single-arm, open-label, dose escalating, repeat-dose study (defined as cycle 1). Eligible patients were enrolled in sequential cohorts to receive one cycle of five weekly subcutaneous injections of atacicept (Merck Serono S.A. – Geneva, Switzerland (an affiliate of Merck KGaA, Darmstadt, Germany) and ZymoGenetics Inc., Seattle, WA, USA) at doses of 2 mg kg^−1^ (cohort 1, 3 patients), 4 mg kg^−1^ (cohort 2, 3 patients), 7 mg kg^−1^ (cohort 3, 3 patients) or 10 mg kg^−1^ (cohort 4, 7 patients). Escalation to the next dose cohort and identification of the maximum tolerated dose (MTD) for atacicept depended on the frequency of dose-limiting toxicities (DLTs) in the preceding cohort and approval by the safety committee.

**Figure 1:**
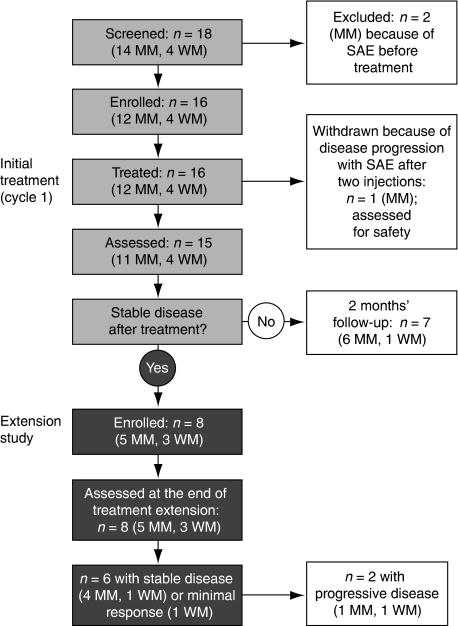
Study design and patient disposition. Abbreviations: MM, multiple myeloma; SAE, serious adverse event; WM, Waldenström's macroglobulinemia.

Patients who completed cycle 1, demonstrated at least stable disease after a 4-week follow-up period and had not experienced any DLTs were eligible to enter the extension study. During the extension study, patients received either two additional cycles of atacicept subcutaneously (five weekly injections), at the same dose as cycle 1, separated each by a 4-week wash-out period (cohorts 1–3) or one continuous cycle of 15 weekly injections of atacicept 10 mg kg^−1^ (cohort 4). After the extension treatment cycles, patients entered a 4-week post-treatment period and a subsequent 2-month follow-up period. Figure 1 summarises the study design and patient disposition.

### Patients

The main study inclusion criteria were: measurable disease, at least one previous regimen of treatment (chemotherapy and/or stem cell transplantation), Eastern Cooperative Oncology Group performance status of 0–2, life expectancy of at least 6 months, adequate hepatic and renal functions (serum aspartate aminotransferase or alanine aminotransferase activity ⩽2.5 times the upper limit of normal (ULN) or serum total bilirubin ⩽1.5 times ULN, creatinine ⩽2 times ULN) and stable haematological parameters (platelet count of ⩾50 000 mm^−3^, haemoglobin concentration of ⩾8 g 100 ml^−1^ and absolute neutrophil count of ⩾1000 mm^−3^) and the willingness to use acceptable methods of contraception during the study and until 3 months after the last dose of atacicept. The main exclusion criteria included: non-secretory myeloma, amyloidosis, active infection, stem cell transplantation in the previous 6 months, receipt of any investigational product, radiation, chemotherapy, biological therapy, immunotherapy or corticosteroids (>10 mg day^−1^) in the previous month, dialysis concurrent to study treatment, previous history of congestive heart failure, significant cardiac ejection fraction abnormalities and presence or history of other types of cancer.

### Safety

Throughout both studies, safety was assessed by adverse event (AE) reports, occurrence of DLTs, direct physical examination and clinical laboratory tests.

Owing to the mechanism of action of atacicept, AEs in the system organ class (SOC) of ‘Infections and infestations’ were closely monitored.

DLTs were defined by using the Common Toxicity Criteria for Adverse Events (Version 3, 12 December 2003). Non-haematological DLTs: (1) any serious AE unless unrelated to the study drug and (2) any grade 3 AE possibly, probably or definitely related to the study drug. Haematological DLTs: (1) any AE of ⩾grade 3 possibly, probably or definitely related to the study drug (except B-cell lymphopenia using the grading scale for haematological toxicity from the National Cancer Institute-Sponsored Working Group Guidelines for Chronic Lymphocytic Leukaemia (Cheson *et al*, 1996), which was an expected effect of BLyS and APRIL depletion; (2) hypersensitivity reactions of ⩾grade 2 (considered dose limiting for an individual); or (3) grade 3 or 4 hypersensitivity reactions reported in multiple patients at the same dose level (considered DLTs for the study). Injection-site reactions were considered as non-haematological events and were evaluated using non-haematological DLT criteria. The assessment of anti-atacicept binding antibodies was carried out before entry into the study and 2–3 months after the last treatment dose.

### Pharmacokinetic assessment

Serum concentrations of free atacicept, atacicept–BLyS complex and total atacicept (free plus atacicept–BLyS complex) were assessed in all patients before injection and weekly after injection during cycle 1, according to previously published methods (Tak *et al*, 2008). Atacicept–APRIL complexes were not assessed in this study.

### Response evaluation

Response evaluation was carried out 4 weeks after treatment cessation following both cycle 1 and the extension study, using published response criteria (Bladé *et al*, 1998; Cheson *et al*, 1999). Magnetic resonance imaging, computerised (axial) tomography scan and/or ultrasound scan (for patients with WM only) were carried out in addition to skeletal X-ray surveys (optional).

### Biological assessments

Pharmacodynamic parameters included concentrations of M-protein (by electrophoresis and immunofixation), *β*2-microglobulin, polyclonal Igs and C-reactive protein (CRP) in serum and free light chain in serum and urine. Bone marrow aspirates were carried out at screening, at the end of treatment and at the end of the 4-week follow up during cycle 1, and at the end of treatment during the extension study (Figure 1). Phenotyping of lymphocyte lineages and myeloma cells was carried out by Esoterix (Mechelen, Belgium) on peripheral blood and bone marrow samples using flow cytometry analysis and the cell-specific antibodies CD3, CD4, CD8, CD14, CD19, CD27, CD38, CD45, CD56, kappa and lambda. Soluble Syndecan-1 (sCD138) was measured in patient sera using an enzyme-linked immunosorbent assay (ELISA) with 8 ng ml^−1^ sensitivity (Diaclone, Besançon, France). Serum concentrations of free BLyS and APRIL were assessed using ELISA methodology (Tak *et al*, 2008) before treatment, but not during treatment, as the presence of atacicept interfered with the assays.

### Data analysis and statistics

The safety population comprised all patients who received at least one injection of atacicept. All efficacy analyses were carried out in the efficacy population, which included all patients who received ⩾4 injections of atacicept during cycle 1. In the extension study, patients who received at least one injection of atacicept were included in the efficacy analyses. Owing to the exploratory nature of this trial, descriptive statistics and graphical representations were used to summarise data.

The statistical significance of the decrease in polyclonal Ig levels after atacicept treatment, of the percentage of myeloma cells in the bone marrow at the end of cycle 1, and of the plasma levels of sCD138 at the end of each cycle, were analysed using Wilcoxon's test for pairs.

## Results

### Safety

Sixteen patients (out of 18 patients screened for participation) entered cycle 1 (Figure 1) and were included in the safety population. Demographic and baseline disease characteristics are shown in Table 1. Treatment with atacicept at the four dose levels was well tolerated both systemically and locally in all patients with MM or WM, during cycle 1 and in all extension schedules, with most AEs being of mild or moderate severity. The MTD of atacicept was not identified, as no DLTs were observed.

All patients experienced at least one AE during the treatment period of cycle 1. The severity of the 57 treatment-emergent AEs reported was as follows: 45 mild, 10 moderate and 2 severe. The 10 (17.5%) AEs of moderate severity that occurred during the treatment period of cycle 1 are listed in Table 2. The 2 (3.5%) severe AEs that occurred during the treatment period of cycle 1 were one event of lung disorder (2 mg kg^−1^) and one of motor dysfunctions (10 mg kg^−1^), both of which were assessed as unrelated to study treatment. The case of severe pneumopathy was reported to have a possible infectious component and the patient was hospitalised and treated with antibiotics. A thoracic scan showed a large tumoral mass with underlying osteolysis. This patient was hospitalised with pneumopathy on two further occasions and treated with antibiotics; sequelae of progressive disease and pleural effusion were noted. The patient with motor dysfunction was withdrawn from treatment after receiving two doses of atacicept. The patient had medullar compression due to myeloma metastasis. Surgery was performed to relieve the compression and the patient was transferred to the haematology unit and discharged after 1 month.

Four patients each experienced one serious AE (pneumopathy, epiploic appendagitis, progression of MM and motor dysfunction) during the cycle 1 treatment period, none of which was considered to be related to atacicept. The patient with progression of MM died during the follow-up period of cycle 1; the cause of death was recorded as MM. No serious AEs were reported during the extension study.

Few events were reported in the SOC ‘Infections and infestations’ during cycle 1 or the extension study; all of these were mild or moderate in severity and none of these events was considered to be related to atacicept treatment. The events in this SOC included upper respiratory and urinary tract infection, gastroenteritis, influenza, rhinitis, nasopharyngitis, fungal infection (one patient reported mild inguinal mycosis, which resolved without intervention) and bronchitis.

None of the 13 patients tested was positive for anti-atacicept binding antibodies at study end.

### Pharmacokinetics

**Figure 2:**
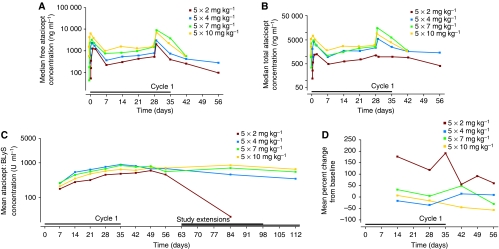
(**A**) The change in median free atacicept concentration during disease evolution in patients with multiple myeloma (MM) or Waldenström's macroglobulinemia (WM). (**B**) The change in median total atacicept (free plus atacicept–BLyS complex) concentration during disease evolution in patients with MM or WM. (**C**) The change in median atacicept–BLyS complex concentration during disease evolution in patients with MM or WM. 1 U ml^−1^ corresponds to 1.82 ng ml^−1^ of atacicept to 0.44 ng ml^−1^ of B-lymphocyte stimulator (BLyS) in a 1 : 3 molar ratio. (**D**) Mean percent of change from baseline in total B cells (CD45^+^/CD19^+^) in patients with MM or WM.

In general, free atacicept, total atacicept and atacicept–BLyS complex behaved consistently across the dose cohorts studied (Figure 2A–C). A mild non-linearity in the pharmacokinetic variables was observed (data not shown), probably because of the mediation of the kinetics of atacicept by its ligands, as reported previously (Munafo *et al*, 2007; Tak *et al*, 2008). There was a slightly higher accumulation of atacicept–BLyS complex and total atacicept compared with that of free atacicept, yielding a moderate accumulation ratio of approximately two for the five consecutive doses in cycle 1.

### Response evaluation

Among the 12 patients with MM who entered the initial treatment phase, 11 completed cycle 1 (patients 1, 2, 3, 5, 7, 8, 11, 13, 14, 17 and 18) and were analysed for response 4 weeks after treatment cessation (efficacy population). Five patients (45%, patients 2, 3, 11, 13 and 18) were progression free and received additional treatment during the extension study (Figure 1 and Table 3). At the end of the extension study, four patients (36%, patients 2, 3, 11 and 18) were progression free. For these four patients with stable disease, all the parameters considered for treatment response, which included *β*2-microglobulin, calcium, albumin, haemoglobin and creatinine levels, and white blood cell count, as well as the M-protein concentrations, remained stable. No new bone lesions were reported in these patients.

All four patients with WM completed cycle 1 (patients 4, 6, 10 and 12) and were analysed for response (efficacy population). Three patients were progression free at the end of cycle 1 (patients 4, 10 and 12; Figure 1 and Table 3). At the end of the extension study, two patients (patients 10 and 12) were progression free (one patient had a minimal response and the other patient had stable disease). No progression of the tumour mass was observed in the two patients who had lymph node involvement at study entry.

### Biological responses

**Figure 3:**
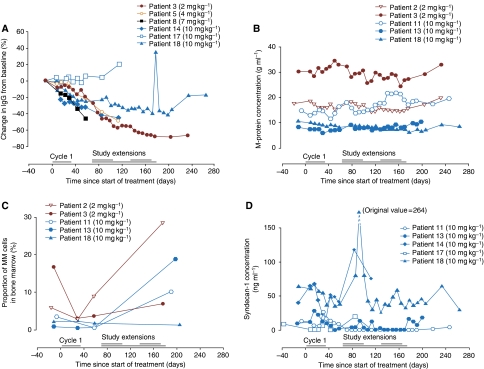
(**A**) The percentage change from baseline in polyclonal immunoglobulin-G (IgG) component throughout cycle 1 and the extension study for patients with multiple myeloma (MM). Patients 1, 2, 7, 11 and 13 from the efficacy population are not shown as they had IgG monoclonal component. (**B**) The change in M-protein concentration (disease specific marker) throughout cycle 1 and the extension study in patients with MM who demonstrated stable disease after cycle 1. (**C**) The change in the proportion of MM cells (CD45dim CD38^+++^) in the bone marrow throughout cycle 1 and the extension study, as determined by flow cytometry analysis, in patients with MM who demonstrated stable disease after cycle 1. (**D**) The change in soluble Syndecan-1 (sCD138) concentration throughout cycle 1 and the extension study in patients with MM who received atacicept 10 mg kg^−1^ (cohort 4). Treatment with atacicept during cycle 1 was the same for all patients. In the extension study, treatment was administered as two cycles of five weekly injections or one cycle of 15 weekly injections.

**Figure 4:**
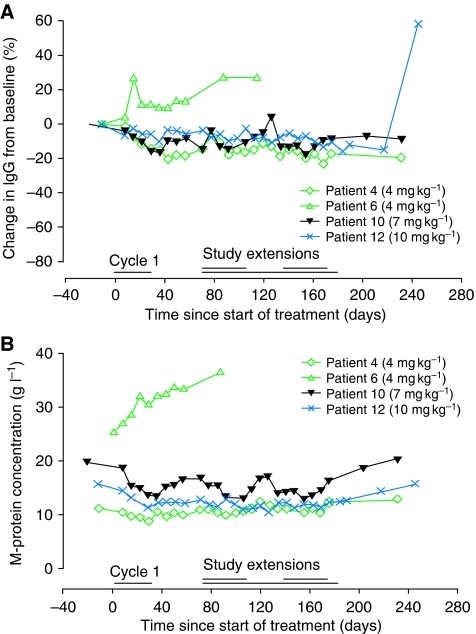
(**A**) Percentage changes from baseline in polyclonal immunoglobulin-G (IgG) component for patients with Waldenström's macroglobulinemia (WM). (**B**) The change in M-protein concentration (disease specific marker) throughout cycle 1 and the extension study in patients with WM.

Polyclonal IgM and IgA were evaluated in patients with IgG MM, polyclonal IgM and IgG in patients with IgA MM and polyclonal IgG and IgA in patients with WM. Consistent with the mechanism of action of atacicept, a significant decrease of the polyclonal Ig isotypes (median decrease 40%, range 10–70%) was seen in 6 out of 11 patients with MM (patients 2, 3, 5, 8, 14 and 18) and 3 out of 4 patients with WM (patients 4, 10 and 12) (*P*⩽0.05) at the end of cycle 1. The magnitude of the reduction appeared to be homogeneous across the three isotypes studied, and was notable across all dose and disease groups. In addition, this reduction lasted until the end of follow up. Representative results for polyclonal IgG are shown in Figure 3A for the six patients with IgA-secreting MM, and in Figure 4A for the four patients with WM. Consistent with the decrease in polyclonal Ig, a dose-dependent reduction in total circulating B cells (CD45^+^ and CD19^+^) was observed (Figure 2D). Neither natural killer cell nor T-lymphocyte subset counts were altered (results not shown).

Five patients with MM had stable or slightly decreased concentrations of M-protein during cycle 1 (patients 2, 3, 11, 13 and 18; Figure 3B). Stabilisation of M-protein concentrations was confirmed in four patients who received extended treatment (patients 2, 3, 11 and 18; Figure 3B). The corresponding kappa and lambda light-chain serum concentrations followed a similar trend (data not shown). Consistent with the stabilisation/reduction in M-protein in five patients, there was a decrease in the percentage of CD45dim CD38^+++^ MM cells in the bone marrow at the end of cycle 1 (*P*⩽0.05, Figure 3C). Furthermore, sCD138 concentration was reduced, most strikingly in patients receiving the highest dose of atacicept who entered the extension study. A decrease in sCD138 concentration was consistently seen at the end of the first treatment cycle in this cohort of patients (*P*⩽0.05, Figure 3D). Two patients with stable disease during the extension study had very low concentrations of sCD138 or levels below the limit of sensitivity of the assay, up to 2 months after treatment termination. This decrease of sCD138 measurement was not due to the interference of atacicept with sCD138 dosage, as atacicept can bind sCD138. Indeed, adding graded concentrations of atacicept in the ELISA did not change sCD138 titration (results not shown). The biological effect of atacicept was more pronounced in patients with WM. Of those who entered the extension study, one patient had a minimal response (decrease of ⩾25% from baseline in M-protein concentration) and a noticeable decrease was seen in the serum M-protein concentrations in the remaining two patients during the first month of treatment and throughout the extension study (Figure 4B). However, the optimal biological dose (defined as a dose leading to >25% decrease in M-protein concentration at day 57 of the first treatment cycle) was not identified for patients with MM or WM.

Atacicept did not affect the inflammatory biomarkers, CRP (range at baseline: 2–400 mg l^−1^) or erythrocyte sedimentation rate (data not shown).

Of the 16 patients tested at baseline, 13 had measurable levels of free APRIL (⩾25 ng ml^−1^). In contrast, only three patients had baseline free BLyS concentrations above the limit of quantification (1.6 ng ml^−1^). In this small number of patients, no correlations were apparent between baseline levels of free APRIL and biological or clinical response criteria.

## Discussion

This is the first full report of atacicept treatment in patients with MM and WM. A preliminary report has previously been presented (Rossi *et al*, 2005). Atacicept at doses of up to 10 mg kg^−1^ was generally well tolerated and had a favourable safety profile. However, because of the small population size and relatively low numbers of AEs, no definitive patterns in the frequency or nature of events could be determined with respect to dose.

One of the primary objectives of cycle 1, the identification of the MTD of atacicept, was not achieved: the maximum dose that could be delivered by subcutaneous injection was 10 mg kg^−1^, and this dose did not elicit DLTs. Similarly, no DLTs were observed using repeated doses of atacicept up to 10 mg kg^−1^ in a study in patients with advanced B-cell lymphomas in which, although atacicept showed biological activity, clinical responses were not observed (Ansell *et al*, 2008). In this study, it is encouraging that, as well as being generally well tolerated up to 10 mg kg^−1^, both biological and clinical responses were observed.

Of particular note, a decrease across all measured polyclonal Ig isotypes was seen in most patients, concomitant with a notable, but incomplete, decline in circulating Ig-producing cells. Atacicept has been associated with dose-dependent reductions in levels of polyclonal Ig isotypes and circulating B cells in patients with rheumatoid arthritis (Tak *et al*, 2008), and systemic lupus erythematosus (Dall’Era *et al*, 2007). B cells mature into plasmablasts in germinal centers of lymphoid organs, these cells then migrate to the bone marrow or mucosa where they differentiate into plasma cells, which can survive for up to 20 years, thus ensuring the continuity of humoral immunity (Hofer *et al*, 2006). The maintenance of the plasma cell pool has been shown to be independent of memory B cells (Ahuja *et al*, 2008). Long-term treatment with anti-CD20 antibody can profoundly deplete B cells and the generation of new plasma cells, without affecting the circulating polyclonal Ig and the pool of plasma cells (Ahuja *et al*, 2008). Thus, the decrease in polyclonal Ig with atacicept suggests that BLyS and APRIL may be critical factors for the long-term survival of plasma cells. This suggestion is supported by data from experiments in mice showing that BCMA activation by BLyS/APRIL is mandatory for plasma cell differentiation (O′Connor *et al*, 2004), and that atacicept treatment can reduce the numbers of both mature B cells and plasma cells (Ramanujam *et al*, 2006; Benson *et al*, 2008). Stabilisation or a slight reduction in M-protein concentration was seen during the first treatment cycle in almost half of the patients with MM, and was maintained in the majority of patients who entered the extension study. This pattern was repeated for corresponding serum concentration profiles of kappa or lambda light chains. Consistent with these serum marker features were noticeable decreases in the percentage of bone marrow CD45dim CD38^+++^ MM cells and circulating levels of sCD138, reflecting the production of sCD138 by these cells in the bone marrow (Klein *et al*, 1999; Mahtouk *et al*, 2007). This is of particular interest, given the proposed role of sCD138 in the accumulation of APRIL in the bone marrow stroma, the binding of APRIL to its receptors and the ensuing mediation of plasma-cell survival (Hendriks *et al*, 2005; Ingold *et al*, 2005; Moreaux *et al*, 2009).

These convergent observations suggest that atacicept inhibits MM cell survival within the bone marrow microenvironment rather than merely altering monoclonal Ig synthesis by MM cells, as predicted from *in vitro* treatment studies using primary myeloma cells (Moreaux *et al*, 2005; Abe *et al*, 2006; Yaccoby *et al*, 2008). In addition, atacicept did not affect the inflammation markers often elevated in B-cell malignancies, suggesting that it does not interfere with the interleukin-6-dependent pathway. The lack of a correlation between free APRIL levels at baseline and clinical or biological effects is unsurprising in this study, as numbers per dose cohort were small and the effects of atacicept showed dose dependence.

The biological effect of atacicept treatment appeared more marked and consistent among patients with WM, with three out of four patients showing a noticeable decrease in M-protein concentration during the first month of treatment. However, a rise in M-protein levels was seen in the fourth patient. A paradoxical rise in M-protein levels has also been observed with rituximab treatment in patients with WM (Treon *et al*, 2004) and has been associated with symptomatic hyperviscosity syndrome. The possibility of a paradoxical rise in serum IgM levels should be taken into consideration in future trials investigating the effects of atacicept in patients with WM.

The relatively low circulating concentrations of BLyS observed at baseline are in contrast with previous reports (Moreaux *et al*, 2004). However, peripheral BLyS concentrations may not reflect the local BLyS production in the bone marrow microenvironment. Increased BLyS production could explain the increased concentration of serum atacicept–BLyS complexes. Indeed, these complexes reflect the BLyS binding load, which may explain why a resumption of disease progression, rather than a rebound phenomenon, was observed after treatment cessation.

Five of the patients with MM who completed the first treatment cycle had stable disease, and four of these maintained stable disease after the extension treatment period. In addition, three of the four patients with WM had stable disease after completion of the first treatment cycle, and two of these had either a minimal response or stable disease after the extension treatment period. These findings, together with the favourable safety profile and evidence of the biological activity of atacicept in this phase-I trial, may justify further investigation. Although significant infection-related AEs have not been associated with atacicept treatment here or in initial studies in other indications (Dall’Era *et al*, 2007; Ansell *et al*, 2008; Tak *et al*, 2008), surveillance for effects on infection should remain a high priority because of the possible association between suppression of polyclonal B cells and infection. Basic scientific investigations should be focused on characterising the role of atacicept in the MM bone-marrow microenvironment, specifically in the sCD138 matrix.

## Figures and Tables

**Table 1:** Demographic and baseline disease characteristics (safety population)

**Characteristic**	**Statistics**	**Overall n=16**
Age, years	Mean (s.d.)	67.9 (6.7)
	Median (range)	70 (51–75)
Sex, *n* (%)	Male	9 (56.2)
	Female	7 (43.8)
Disease type at diagnosis, *n* (%)	MM	12 (75.0)
	WM	4 (25.0)
Disease stage at diagnosis for patients with MM, *n* (%)^a^	Stage I	2 (16.7)
	Stage II	4 (33.3)
	Stage III	6 (50.0)
Disease status at entry, *n* (%)	Refractory	5 (31.3)
	Relapsed	11 (68.7)
*β*2-microglobulin at baseline (mgl^−1^)	Mean (s.d.)	2.7 (1.1)
Type of Ig for patients with MM, *n* (%)^a^	IgA	6 (50.0)
	IgG	5 (41.7)
	IgD	1 (8.3)
Type of M-protein for patients with MM, *n* (%)^a^	Kappa	7 (58.3)
	Lambda	5 (41.7)
Serum M-protein concentration (gl^−1^)		
	Median (range)	15.6 (2.6–41.8)
Bone marrow plasma cell cytogenic abnormalities, *n* (%)	Yes	6 (37.5)
Number of previous lines of anti-tumour	0	0 (0.0)
treatments, *n* (%)	1	2 (12.5)
	2	6 (37.5)
	3	3 (18.7)
	⩾4	5 (31.3)
Transplant history for patients with MM, *n* (%)^a^	No	3 (25.0)
	Yes	9 (75.0)

Abbreviations: Ig=immunoglobulin; MM=multiple myeloma; s.d.=standard deviation; WM=Waldenström's macroglobulinemia.

^a^*n*=12.

**Table 2:** Treatment-emergent adverse events of moderate severity occurring during the treatment period of cycle 1, by dose regimen (safety population)

**Dose cohort**	**Treatment-emergent adverse events of moderate severity**
2 mg kg^−1^ (*n*=3)	None
4 mg kg^−1^ (*n*=3)	1 haematoma
7 mg kg^−1^ (*n*=3)	1 bone pain; 1 epiploic appendagitis; and 1 multiple myeloma
10 mg kg^−1^ (*n*=7)	2 arthralgia (in 1 patient); 1 back pain, 1 asthenia; 1 pain; and 1 fungal infection

**Table 3:** Clinical responses in patients with multiple myeloma (MM) and Waldenström's macroglobulinemia (WM) who completed the initial treatment period in cycle 1

**Patient number**	**Dose mg kg^−1^**	**MM isotype**	**Cycle 1**	**Extension study^a^**
*Patients with MM*				
1	2	IgG kappa IIIA	Progressive disease	
2	2	IgG lambda IIA	Stable disease	Stable disease
3	2	IgA lambda IIIA	Stable disease	Stable disease
5	4	IgD lambda IA	Progressive disease	
7	7	IgG lambda IA	Progressive disease	
8	7	IgA lambda IIIA	Progressive disease	
11	10	IgG lambda IIA	Stable disease	Stable disease
13	10	IgG kappa IIIA	Stable disease	Progressive disease
14	10	IgA kappa IIIA	Progressive disease	
17	10	IgA kappa IIIA	Progressive disease	
18	10	IgA kappa IIA	Stable disease	Stable disease
				
*Patients with WM*				
4	4	NA	Stable disease	Progressive disease
6	4	NA	Progressive disease	
10	7	NA	Stable disease	Stable disease
12	10	NA	Stable disease	Minimal response

Abbreviations: Ig=immunoglobulin; NA=not applicable.

a Patients who completed cycle 1, demonstrated at least stable disease after a 4-week follow-up period and had not experienced any dose-limiting toxicity were eligible to enter the extension study.

## References

[bib1] Abe M, Kido S, Hiasa M, Nakano A, Oda A, Amou H, Matsumoto T (2006) BAFF and APRIL as osteoclast-derived survival factors for myeloma cells: a rationale for TACI-Fc treatment in patients with multiple myeloma. Leukemia 20: 1313–1315 16617317 10.1038/sj.leu.2404228

[bib2] Ahuja A, Anderson SM, Khalil A, Shlomchik MJ (2008) Maintenance of the plasma cell pool is independent of memory B cells. Proc Natl Acad Sci USA 105: 4802–4807 18339801 10.1073/pnas.0800555105 PMC2290811

[bib3] Ansell SM, Witzig TE, Inwards DJ, Porrata LF, Ythier A, Ferrande L, Nestorov I, Devries T, Dillon SR, Hausman D, Novak AJ (2008) Phase I clinical study of atacicept in patients with relapsed and refractory B-cell non-Hodgkin's lymphoma. Clin Cancer Res 14: 1105–1110 18281543 10.1158/1078-0432.CCR-07-4435

[bib4] Benson MJ, Dillon SR, Castigli E, Geha RS, Xu S, Lam KP, Noelle RJ (2008) Cutting edge: the dependence of plasma cells and independence of memory B cells on BAFF and APRIL. J Immunol 180: 3655–3659 18322170 10.4049/jimmunol.180.6.3655

[bib5] Bladé J, Lopez-Guillermo A, Bosch F, Cervantes F, Reverter JC, Montserrat E, Rozman C (1994) Impact of response to treatment on survival in multiple myeloma: results in a series of 243 patients. Br J Haemat 88: 117–121 7803233 10.1111/j.1365-2141.1994.tb04986.x

[bib6] Bladé J, Samson D, Reece D, Apperley J, Bjorkstrand B, Gahrton G, Gertz M, Giralt S, Jagannath S, Vesole D (1998) Criteria for evaluating disease response and progression in patients with multiple myeloma treated by high-dose therapy and haemopoietic stem cell transplantation. Myeloma Subcommittee of the EBMT. European Group for Blood and Marrow Transplant. Br J Haemat 102: 1115–1123 9753033 10.1046/j.1365-2141.1998.00930.x

[bib7] Cheson BD, Bennett JM, Grever M, Kay N, Keating MJ, O′Brien S, Rai KR (1996) National Cancer Institute-sponsored Working Group guidelines for chronic lymphocytic leukemia: revised guidelines for diagnosis and treatment. Blood 87: 4990–4997 8652811

[bib8] Cheson BD, Horning SJ, Coiffier B, Shipp MA, Fisher RI, Connors JM, Lister TA, Vose J, Grillo-Lopez A, Hagenbeek A, Cabanillas F, Klippensten D, Klippensten D, Hiddemann W, Castellino R, Harris NL, Armitage JO, Carter W, Hoppe R, Canellos GP (1999) Report of an international workshop to standardize response criteria for non-Hodgkin's lymphomas. NCI Sponsored International Working Group. J Clin Oncol 17: 1244 10561185 10.1200/JCO.1999.17.4.1244

[bib9] Dall’Era M, Chakravarty E, Wallace D, Genovese M, Weisman M, Kavanaugh A, Kalunian K, Dhar P, Vincent E, Pena-Rossi C, Wofsy D (2007) Reduced B lymphocyte and immunoglobulin levels after atacicept treatment in patients with systemic lupus erythematosus: results of a multicenter, phase Ib, double-blind, placebo-controlled, dose-escalating trial. Arthritis Rheum 56: 4142–4150 18050206 10.1002/art.23047

[bib10] Dimopoulos MA, Gertz MA, Kastritis E, Garcia-Sanz R, Kimby EK, Leblond V, Fermand JP, Merlini G, Morel P, Morra E, Ocio EM, Owen R, Ghobrial IM, Seymour J, Kyle RA, Treon SP (2009) Update on treatment recommendations from the Fourth International Workshop on Waldenström's Macroglobulinemia. J Clin Oncol 27: 120–126 19047284 10.1200/JCO.2008.17.7865

[bib11] Dimopoulos MA, Panayiotidis P, Moulopoulos LA, Sfikakis P, Dalakas M (2000) Waldenstrom's macroglobulinemia: clinical features, complications, and management. J Clin Oncol 18: 214–226 10623712 10.1200/JCO.2000.18.1.214

[bib12] Elsawa SF, Novak AJ, Grote DM, Ziesmer SC, Witzig TE, Kyle RA, Dillon SR, Harder B, Gross JA, Ansell SM (2006) B-lymphocyte stimulator (BLyS) stimulates immunoglobulin production and malignant B-cell growth in Waldenstrom macroglobulinemia. Blood 107: 2882–2888 16304043 10.1182/blood-2005-09-3552 PMC1895377

[bib13] Ferlay J, Bray F, Pisani P, Parkin DM (2004) GLOBOCAN 2002: Cancer incidence, mortality and prevalence worldwide. IARCPress: Lyon

[bib14] Gras MP, Laabi Y, Linares-Cruz G, Blondel MO, Rigaut JP, Brouet JC, Leca G, Haguenauer-Tsapis R, Tsapis A (1995) BCMAp: an integral membrane protein in the Golgi apparatus of human mature B lymphocytes. Int Immunol 7: 1093–1106 8527407 10.1093/intimm/7.7.1093

[bib15] Gross JA, Johnston J, Mudri S, Enselman R, Dillon SR, Madden K, Xu W, Parrish-Novak J, Foster D, Lofton-Day C, Moore M, Littau A, Grossman A, Haugen H, Foley K, Blumberg H, Harrison K, Kindsvogel W, Clegg CH (2000) TACI and BCMA are receptors for a TNF homologue implicated in B-cell autoimmune disease. Nature 404: 995–999 10801128 10.1038/35010115

[bib16] Gross JA, Dillon SR, Mudri S, Johnston J, Littau A, Roque R, Rixon M, Schou O, Foley KP, Haugen H, McMillen S, Waggie K, Schreckhise RW, Shoemaker K, Vu T, Moore M, Grossman A, Clegg CH (2001) TACI-Ig neutralizes molecules critical for B cell development and autoimmune disease: impaired B cell maturation in mice lacking BLyS. Immunity 15: 289–302 11520463 10.1016/s1074-7613(01)00183-2

[bib17] Harousseau JL, Moreau P (2007) Role of bone marrow transplantation in the disease pathway of myeloma. J Natl Compr Canc Netw 5: 163–169 17335685 10.6004/jnccn.2007.0016

[bib18] Hendriks J, Planelles L, de Jong-Odding J, Hardenberg G, Pals ST, Hahne M, Spaargaren M, Medema JP (2005) Heparan sulfate proteoglycan binding promotes APRIL-induced tumor cell proliferation. Cell Death Differ 12: 637–648 15846369 10.1038/sj.cdd.4401647

[bib19] Hofer T, Muehlinghaus G, Moser K, Yoshida T, E Mei H, Hebel K, Hauser A, Hoyer B, E OL, Dorner T, Manz RA, Hiepe F, Radbruch A (2006) Adaptation of humoral memory. Immunol Rev 211: 295–302 16824136 10.1111/j.0105-2896.2006.00380.x

[bib20] Ingold K, Zumsteg A, Tardivel A, Huard B, Steiner QG, Cachero TG, Qiang F, Gorelik L, Kalled SL, Acha-Orbea H, Rennert PD, Tschopp J, Schneider P (2005) Identification of proteoglycans as the APRIL-specific binding partners. J Exp Med 201: 1375–1383 15851487 10.1084/jem.20042309 PMC2213192

[bib21] Klein B, Li XY, Lu ZY, Jourdan M, Tarte K, Brochier J, Claret E, Wijdenes J, Rossi JF (1999) Activation molecules on human myeloma cells. Curr Top Microbiol Immunol 246: 335–341 10396073 10.1007/978-3-642-60162-0_41

[bib22] Mahtouk K, Hose D, Raynaud P, Hundemer M, Jourdan M, Jourdan E, Pantesco V, Baudard M, De Vos J, Larroque M, Moehler T, Rossi JF, Rème T, Goldschmidt H, Klein B (2007) Heparanase influences expression and shedding of syndecan-1, and its expression by the bone marrow environment is a bad prognostic factor in multiple myeloma. Blood 109: 4914–4923 17339423 10.1182/blood-2006-08-043232 PMC2268882

[bib23] Moreaux J, Legouffe E, Jourdan E, Quittet P, Reme T, Lugagne C, Moine P, Rossi JF, Klein B, Tarte K (2004) BAFF and APRIL protect myeloma cells from apoptosis induced by interleukin 6 deprivation and dexamethasone. Blood 103: 3148–3157 15070697 10.1182/blood-2003-06-1984 PMC2387243

[bib24] Moreaux J, Cremer FW, Reme T, Raab M, Mahtouk K, Kaukel P, Pantesco V, De Vos J, Jourdan E, Jauch A, Legouffe E, Moos M, Fiol G, Goldschmidt H, Rossi JF, Hose D, Klein B (2005) The level of TACI gene expression in myeloma cells is associated with a signature of microenvironment dependence versus a plasmablastic signature. Blood 106: 1021–1023 15827134 10.1182/blood-2004-11-4512 PMC2408610

[bib25] Moreaux J, Hose D, Jourdan M, Reme T, Hundemer M, Moos M, Robert N, Moine P, De Vos J, Goldschmidt H, Klein B (2007) TACI expression is associated with a mature bone marrow plasma cell signature and C-MAF overexpression in human myeloma cell lines. Haematologica 92: 803–811 17550853 10.3324/haematol.10574 PMC2789280

[bib26] Moreaux J, Sprynski AC, Dillon SR, Mahtouk K, Jourdan M, Ythier A, Moine P, Robert N, Jourdan E, Rossi JF, Klein B (2009) APRIL and TACI interact with syndecan-1 on the surface of multiple myeloma cells to form an essential survival loop. Eur J Haematol 83: 119–129 19456850 10.1111/j.1600-0609.2009.01262.x

[bib27] Munafo A, Priestley A, Nestorov I, Visich J, Rogge M (2007) Safety, pharmacokinetics and pharmacodynamics of atacicept in healthy volunteers. Eur J Clin Pharmacol 63: 647–656 17473917 10.1007/s00228-007-0311-7

[bib28] Novak AJ, Darce JR, Arendt BK, Harder B, Henderson K, Kindsvogel W, Gross JA, Greipp PR, Jelinek DF (2004) Expression of BCMA, TACI, and BAFF-R in multiple myeloma: a mechanism for growth and survival. Blood 103: 689–694 14512299 10.1182/blood-2003-06-2043

[bib29] O′Connor BP, Raman VS, Erickson LD, Cook WJ, Weaver LK, Ahonen C, Lin LL, Mantchev GT, Bram RJ, Noelle RJ (2004) BCMA is essential for the survival of long-lived bone marrow plasma cells. J Exp Med 199: 91–98 14707116 10.1084/jem.20031330 PMC1887725

[bib30] Ramanujam M, Wang X, Huang W, Liu Z, Schiffer L, Tao H, Frank D, Rice J, Diamond B, Yu KO, Porcelli S, Davidson A (2006) Similarities and differences between selective and nonselective BAFF blockade in murine SLE. J Clin Invest 116: 724–734 16485042 10.1172/JCI26385 PMC1366500

[bib31] Rossi J-F, Moreaux J, Rose M, Picard M, Ythier A, Rossier C, Sievers E, Klein BA (2005) Phase I/II study of atacicept (TACI-Ig) to neutralize APRIL and BLyS in patients with refractory or relapsed multiple myeloma (MM) or active previously treated Waldenstrom's macroglobulinemia (WM). Blood (ASH Annual Meeting Abstracts) 106: 2566

[bib32] Schneider P, MacKay F, Steiner V, Hofmann K, Bodmer JL, Holler N, Ambrose C, Lawton P, Bixler S, Acha-Orbea H, Valmori D, Romero P, Werner-Favre C, Zubler RH, Browning JL, Tschopp J (1999) BAFF, a novel ligand of the tumor necrosis factor family, stimulates B cell growth. J Exp Med 189: 1747–1756 10359578 10.1084/jem.189.11.1747 PMC2193079

[bib33] Tai YT, Li XF, Breitkreutz I, Song W, Neri P, Catley L, Podar K, Hideshima T, Chauhan D, Raje N, Schlossman R, Richardson P, Munshi NC, Anderson KC (2006) Role of B-cell-activating factor in adhesion and growth of human multiple myeloma cells in the bone marrow microenvironment. Cancer Res 66: 6675–6682 16818641 10.1158/0008-5472.CAN-06-0190

[bib34] Tak P, Thurlings R, Rossier C, Nestorov I, Dimic A, Mircetic V, Rischmueller M, Nasonov E, Shmidt E, Emery P, Munafo A (2008) Atacicept in patients with rheumatoid arthritis: Results of a multicenter, phase Ib, double-blind, placebo-controlled, dose-escalating, single- and repeated-dose study. Arthritis and Rheum 58: 61–72 18163485 10.1002/art.23178

[bib35] Thompson JS, Bixler SA, Qian F, Vora K, Scott ML, Cachero TG, Hession C, Schneider P, Sizing ID, Mullen C, Strauch K, Zafari M, Benjamin CD, Tschopp J, Browning JL, Ambrose C (2001) BAFF-R, a newly identified TNF receptor that specifically interacts with BAFF. Science 293: 2108–2111 11509692 10.1126/science.1061965

[bib36] Treon SP, Branagan AR, Hunter Z, Santos D, Tournhilac O, Anderson KC (2004) Paradoxical increases in serum IgM and viscosity levels following rituximab in Waldenström's macroglobulinemia. Ann Oncol 15: 1481–1483 15367407 10.1093/annonc/mdh403

[bib37] Treon SP, Patterson CJ, Kimby E, Stone MJ (2009) Advances in the biology and treatment of Waldenström's macroglobulinemia: a report from the 5th International Workshop on Waldenström's Macroglobulinemia, Stockholm, Sweden. Clin Lymphoma Myeloma 9: 10–15 10.3816/CLM.2009.n.001 19362961

[bib38] Vitolo U, Ferreri AJ, Montoto S (2008) Lymphoplasmacytic lymphoma-Waldenstrom's macroglobulinemia. Crit Rev Oncol Hematol 67: 172–185 18499469 10.1016/j.critrevonc.2008.03.008

[bib39] Von Bulow GU, Bram RJ (1997) NF-AT activation induced by a CAML-interacting member of the tumor necrosis factor receptor superfamily. Science 278: 138–141 9311921 10.1126/science.278.5335.138

[bib40] Yaccoby S, Pennisi A, Li XY, Dillon SR, Zhan F, Barlogie B, Shaughnessy JDJ (2008) Atacicept (TACI-Ig) inhibits growth of TACI(high) primary myeloma cells in SCID-hu mice and in coculture with osteoclasts. Leukemia 22: 406–413 18046446 10.1038/sj.leu.2405048 PMC2771378

